# Genito-Urinary Surgery

**Published:** 1905-02-25

**Authors:** 


### GENITO-URINARY SURGERY.

(*Continued from page* 370.)

**The Treatment of Syphilis.**—Lawrence T. Royster (Norfolk, Va.),[6] has described the methods he uses in the treatment of the acute stage of syphilis, especially with regard to the hypodermic administration of mercury. He states that no man should ever attempt to diagnose syphilis from the initial lesion alone and that the physician who commences treatment before the appearance of the eruption does himself great injustice and his patient irreparable wrong. During the period of waiting for the eruption the patient is sent to a dentist to have tartar removed, cavities filled and snags drawn. Dental treatment is imperative because a foul mouth is in constant danger of salivation. A slightly astringent mouth wash is to be used at this time. The patient is examined frequently from head to foot for the appearance of an eruption and for glandular enlargement. When the diagnosis is established treatment with mercury is begun. The author objects to giving mercury by the mouth because it "is almost necessarily going to upset some portion of the alimentary canal," and when there is diarrhœa or griping it shows non-absorption of the drug, and it is doubtful if the administration of opium promotes absorption or merely stops the griping. Mercury by the mouth is apt not to be taken regularly by the patient, and it acts slowly. The method of inunction is quick in action and sure, and does not cause digestive disturbances. It is, on the other hand, troublesome and disagreeable. It is an excellent form of treatment when administered by an expert rubber. The advantages of the hypodermic method of treatment are that it very rarely causes digestive disturbances, it is rapid in action, the trouble to patient and doctor is reduced to a minimum, the exact amount of mercury the patient is receiving is known at all times, and neglect of treatment is detected, for the physician gives the injection. The author uses a solution of bichloride of mercury with an equal part of common salt, and makes the injection into the buttocks. As to dosage, the author's routine is as follows:—Begin with 5 minims of a two per cent. solution and increase up to 10 or 12 minims and then change to 5 minims of a four per cent. solution and increase up to about 10 minims, and make this the maximum dose. The use of this concentrated solution has reduced the pain and induration to a minimum. If the eruption is well out the injection is given daily until the eruption has disappeared; when the eruption is slight or seen very early, every other day suffices. The intervals are gradually lengthened until by the end of ten months one injection each week is given.

**The Treatment of Rectal Prolapse by the Submucous Injection of Paraffin.**—Arthur H. Burgess[7] has employed this method of treatment in prolapse of the rectum in 19 cases. The paraffin he uses has a melting point of 111°, and can be obtained sterilised in small bottles. It is melted by placing the bottles in water of a temperature of 120° Fahr., and the syringe is similarly warmed in water before and after it has been filled. The author's technique is the following:—Under anæsthesia the patient is placed in the lithotomy position, and the prolapse drawn downwards as much as possible. The apex of the prolapse is seized in artery forceps at three equidistant places, two anterior and one posterior, and by traction the mucous membrane is raised into three ridges forming an equilateral triangle. An injection of two or three cubic centimètres of paraffin is then made into the centre of each ridge. The swelling produced narrows this lumen of the bowel to a tri-radial slit. This portion of the bowel is reduced, and the process repeated about $1\frac{1}{2}$ inches lower down, but this time two of the forceps are posterior and one anterior. As a rule three tiers of injection are sufficient. To prevent the prolapse descending before the paraffin has set, a stout silkworm-gut suture is passed through the buttocks and tied firmly over a pad of gauze. This suture is removed in 24 hours. The patient is kept in bed for four or five days. The ages of the patients treated ranged from three to 48 years. The result of the operation was extremely satisfactory. Two of the cases had previously been treated by linear cauterisation, and two by excision of the lower part of the rectum. All the cases, with one exception, were under observation for at least two months and many for much longer periods. The action of the paraffin must be purely mechanical at first. It impairs the flexibility of the bowel wall which permits the prolapse. Possible dangers of paraffin injections are sepsis and pulmonary embolism, but in the author's cases no ill effects were observed. The advantages of this method of treatment are its simplicity and safety, the absence of prolonged after treatment and the degree of immediate and probably permanent relief it gives. It is suggested that prolapsus in an artificial anus might be similarly treated.

[6] Med. Rec, Aug. 13, 1904, p. 254. [7] Lancet, Sept. 10, 1904, p. 759.

